# Inflammation–fibrosis coupling in androgenetic alopecia: evidence and mechanistic hypotheses for progressive hair follicle miniaturization

**DOI:** 10.3389/fimmu.2026.1960477

**Published:** 2026-09-30

**Authors:** Yi Li, Xiaoyue Wan, Yun Geng, Wenlong Zhu, Hui Xu, Yuyun Xiong, Yunwen Zheng, Yumei Li

**Affiliations:** 1 Institute of Regenerative Medicine, and Department of Dermatology, Affiliated Hospital of Jiangsu University, Jiangsu University, Zhenjiang, Jiangsu, China; 2 Guangdong Provincial Key Laboratory of Large Animal Models for Biomedicine, and South China Institute of Large Animal Models for Biomedicine, Wuyi University, Jiangmen, Guangdong, China; 3 School of Pharmacy and Food Engineering, Wuyi University, Jiangmen, Guangdong, China; 4 Department of Medical and Life Sciences, Faculty of Pharmaceutical Sciences, Tokyo University of Science, Chiba, Japan; 5 Center for Stem Cell Biology and Regenerative Medicine, Institute of Medical Science, The University of Tokyo, Tokyo, Japan

**Keywords:** androgenetic alopecia, dermal papilla, extracellular matrix, fibroimmune crosstalk, hair follicle miniaturization, perifollicular fibrosis, perifollicular inflammation

## Abstract

Androgenetic alopecia (AGA) is primarily explained by genetic susceptibility and androgen-dependent signaling in dermal papilla cells. This mini-review examines whether low-grade, regionally heterogeneous perifollicular inflammation interacts with extracellular matrix remodeling as a complementary component of AGA pathobiology, without implying an autoimmune mechanism. Human AGA scalp studies report upper-follicular and perifollicular CD4+ lymphocytes, mast cells, immune-enriched transcriptional states, connective tissue sheath abnormalities, and fibrosis in subsets of patients. Patient-derived cells, *ex vivo* follicles, animal models, and cross-disease studies provide additional support for transforming growth factor-β, interleukin-6, prostaglandin D2, Wnt antagonism, oxidative stress, mitochondrial dysfunction, and cellular senescence. These signals could recruit or retain immune cells and shift dermal sheath cells or perifollicular fibroblasts toward matrix-producing and contractile states. Collagen accumulation, altered matrix turnover, stiffness, vascular restriction, and impaired epithelial–mesenchymal crosstalk may then reduce regenerative plasticity and stabilize miniaturization. Human evidence is strongest for androgen-dependent follicular stress and regional inflammatory or fibrotic changes. Specific immune-to-fibroblast circuits, mechanotransduction pathways, and their temporal sequence are supported mainly by experimental models or cross-disease evidence. Spatially resolved inflammation–fibrosis endotypes may help explain variation in treatment response and guide biomarker-enriched trials, but would not by themselves imply an autoimmune pathogenesis.

## 1 Introduction

Androgenetic alopecia (AGA) is the most common type of progressive, non-scarring scalp hair loss. Genetic predisposition, local androgen metabolism, androgen receptor signaling, and the response of dermal papilla cells to dihydrotestosterone (DHT) remain the best-supported framework for explaining the transformation of terminal hair follicles into vellus-like follicles (1). Consequently, evidence-based treatments primarily focus on 5α-reductase inhibition and minoxidil; however, gains in hair density vary significantly among patients, therapeutic plateaus are common, and follicles that have undergone miniaturization for years often fail to fully recover (2). The androgen-centered model remains essential but may not fully explain why long-standing miniaturized follicles respond incompletely to treatment.

Androgen-responsive dermal papilla cells influence the epithelium through paracrine signaling, placing the follicle within a broader epithelial–mesenchymal microenvironment rather than treating it as a passive DHT target (3). Classic scalp histology described perifollicular T lymphocytes, macrophage-like cells, mast-cell activation, and collagen remodeling in male-pattern alopecia; Jaworsky et al. characterized these infiltrates in 1992, and Mahé et al. subsequently termed the process “microinflammation” (4, 5). Peripilar trichoscopic signs were later associated with inflammatory infiltrates and fibrosis (6). These observations indicate that immune and stromal changes accompany AGA, but do not establish that they initiate the disease.

Causal direction remains the principal uncertainty in this framework. Inflammation may precede visible miniaturization in a susceptible scalp, arise secondarily from repeated cycling and follicular stress, or participate bidirectionally at different disease stages; collagen accumulation may likewise constrain regeneration or record prior tissue turnover. Accordingly, this review distinguishes direct human association, experimental mechanism, and cross-disease inference rather than treating them as equivalent. In a retrospective referral cohort, alopecic and clinically non-alopecic scalp frequently showed perinfundibular/periisthmic lymphocytes, CD117-positive mast cells, predominantly CD4-positive infiltrates, and fibrosis, but the lack of external healthy controls and enrichment for complex or treatment-refractory cases precluded prevalence estimates (7). Histopathological overlap among inflammatory AGA, seborrheic inflammation, and patterned fibrosing alopecia further complicates diagnosis (8). The hypothesis considered here is that persistent microinflammation and stromal remodeling may form a feed-forward axis that stabilizes miniaturization in an inflammatory–fibrotic subset of AGA.

## 2 Microinflammation in the androgen-sensitive hair follicle

Microinflammation is anatomically heterogeneous rather than a uniform follicular state. The strongest human histological evidence localizes CD4+ lymphocytes and CD117-positive mast cells to the infundibular/isthmic and upper perifollicular compartments (4, 5, 7). The bulge is immunologically important because it contains epithelial stem cells, but a sustained destructive bulge-directed infiltrate has not been established in uncomplicated AGA. In the dermal sheath and perifollicular dermis, mast cells, macrophage-like cells, fibroblasts, vascular-associated stromal cells, and a contracted connective tissue sheath may participate in matrix remodeling (7, 9). At the bulb and dermal papilla, the best-supported events are androgen-responsive epithelial–mesenchymal stress signals; transcriptomic NK-cell and CD8+ T-cell signatures have been reported, but their precise spatial localization and temporal relationship to miniaturization remain uncertain (10). These regional differences matter because upper-follicular inflammation may alter barrier or sebaceous biology, sheath-centered inflammation may affect tissue mechanics, and bulb/dermal-papilla signaling may more directly alter anagen growth.

A recent AGA fibrosis review places microinflammation within progressive remodeling (11). Bulk and targeted sequencing identify NK-cell and CD8+ T-cell signals in affected scalp (10), whereas single-cell data identify connective tissue sheath contraction and altered stromal communication (9). Together, these findings support region-resolved analysis but do not identify a common initiating lesion (Section 1).

Potential triggers may originate from various anatomical levels. The sebum-rich infundibular skin is exposed to microbial products, ultraviolet (UV) radiation damage, mechanical stress from grooming, and oxidized lipids. Cross-sectional studies of the bacterial and fungal microbiomes in AGA have reported differences in microbial composition (12). A subsequent study combined human scalp analysis with experiments involving mice and hair follicle cells, though effects were not consistently observed across species (13). Research on dandruff has provided supportive—albeit not AGA-specific—evidence indicating that inflammation of the skin surface can coexist with epidermal T-cell infiltration and a reduction in hair follicle immune privilege (14). Thus, while microbiome associations may represent plausible regulatory factors, sebum levels, seborrheic dermatitis, prior treatment exposure, and sampling depth remain significant confounding variables that few studies have adequately controlled for.

The cellular composition is also region-dependent. Histology most strongly supports CD4+ T cells and mast cells in upper perifollicular AGA infiltrates (7), whereas cytotoxic lymphocyte signals mainly derive from transcriptomic studies of advanced disease (10). A Mendelian randomization analysis linked circulating immune and protein traits, including regulatory T-cell markers, to AGA risk, but the genetic instruments cannot localize those effects to scalp compartments (15). CXCL12–CXCR4 signaling may regulate immune-cell retention and stromal crosstalk across alopecias (16). At physiological baseline, IL-15 signaling in human follicles can help maintain immune privilege (17). By contrast, pathological IL-15/IL-15Rβ signaling in alopecia areata supports cytotoxic T-cell activation and disease propagation in human transcriptomic and mouse-model data (18). These context-dependent effects should not be extrapolated directly to AGA.

Current evidence does not justify classifying AGA as an autoimmune alopecia. No disease-specific autoantibody has been established for AGA, and no reproducible HLA association has been shown to define the disorder; these absences do not exclude a modulatory role for local immune signaling. Reported immune infiltrates are generally low-grade, heterogeneous, and concentrated in upper-follicular or perifollicular compartments, whereas a sustained destructive bulb- or bulge-directed attack has not been established in uncomplicated AGA (4, 5, 7, 10). Follicular units persist, and AGA is clinically non-scarring; human single-cell data indicate preservation of the hair-follicle stem-cell compartment despite abnormal connective-tissue-sheath contraction (1, 9). This pattern differs from the cytotoxic immune process characterized in alopecia areata (18) and from the irreversible follicular destruction that defines cicatricial alopecia. Accordingly, immune signaling is better interpreted as a potential modifier or amplifier of androgen-dependent disease than as proof of autoimmune etiology.

Mast cells warrant particular attention; their proximity to blood vessels, nerves, epithelium, and fibroblasts allows mediators such as histamine, tryptase, chymase, TNF, IL-6, and TGF-β to simultaneously influence inflammation and tissue repair. Cells of the macrophage lineage can either promote the resolution of injury or sustain matrix remodeling depending on local signals; a simplistic M1/M2 classification tends to obscure rather than clarify this plasticity. While neutrophils and complement components may mechanistically participate when the barrier is compromised, there is scant evidence of their sustained involvement in uncomplicated AGA. Inflammation of the sebaceous gland may alter the microenvironment of the upper hair follicle without necessarily affecting the hair bulb.

Macrophages, antigen-presenting cells, Langerhans cells, neutrophils, and complement components may influence whether a transient response resolves or shifts toward profibrotic remodeling, but direct AGA evidence is limited and does not establish sustained participation in uncomplicated disease. Their roles should be tested in models that preserve follicular architecture and anatomical compartments. New *in vitro* AGA organ models (19) and immunocompetent hair follicle microphysiological systems (20) may help separate DHT-driven stress from barrier, microbial, and immune effects. These findings are more consistent with microinflammation as a variable modifier of disease and treatment response than as a universal initiating lesion.

## 3 Fibrotic remodeling around miniaturizing follicles

Perifollicular fibrosis differs from the concentric scarring of primary cicatricial alopecia, in which the bulge is destroyed and the follicle is replaced. In AGA, follicles persist, but collagen compaction around the upper follicle, connective tissue sheath alterations, and changes in the dermal papilla niche may reduce regenerative plasticity. A systematic evidence synthesis places fibroblasts at the center of this proposed transition, while emphasizing that the evidence combines human, animal, and *in vitro* studies (21). Human AGA single-cell data show a contracted dermal sheath state (9), and a DKK3–CKAP4 fibroimmune circuit associated with miniaturization (22) further supports an active stromal contribution.

Relevant stromal cell populations include dermal papilla cells, dermal sheath cells, perifollicular fibroblasts, vascular-associated stromal cells, and dermal interstitial fibroblasts. Within the follicular microenvironment, the collagenous matrix regulates growth factor availability, chemokine gradients, cell migration, and tissue mechanics (23). Consequently, TGF-β-SMAD signaling, connective tissue growth factor (CTGF/CCN2), lysyl oxidase (LOX), collagens type I and III, fibronectin, and imbalances between matrix metalloproteinases and their tissue inhibitors are all candidate contributing factors. Reviews of skin fibrosis support the involvement of epithelial-mesenchymal transition-like programs and fibroblast activation (24), while broader research on TGF-β demonstrates extensive crosstalk with JAK-STAT, Wnt, Notch, and growth factor pathways (25). Although keloid biology lends mechanistic plausibility to these pathways, the keloid phenotype—characterized by excessive scarring—should not be directly extrapolated to AGA, given the relatively subtle stromal changes observed in the latter (26).

Matrix stiffness may be as important as matrix quantity. Integrin–FAK signaling and YAP/TAZ transduce mechanical loads into programs controlling contractility and matrix production (27). Lumican and other matrix components influence fibroblast differentiation in skin fibrosis (28). In miniaturized follicles, analogous processes could restrict dermal papilla expansion, alter capillary geometry, and impair epithelial–mesenchymal crosstalk. Whether AGA remodeling is reversible remains unknown: early matrix turnover may be modifiable, whereas long-term cross-linking and sheath contraction may be less responsive. Fibrosis in AGA is therefore better viewed as a spectrum of regenerative limitation than an all-or-none state.

Various markers can distinguish active remodeling from established scarring. Newly synthesized procollagen, fibronectin, periostin, α-smooth muscle actin, LOX activity, and altered MMP/TIMP ratios indicate dynamic interstitial processes, whereas densely cross-linked collagen, loss of follicular units, and persistent dermal sheath contraction suggest enduring structural changes. Vascular remodeling also warrants attention: reduced capillary compliance and increased diffusion distances may limit the delivery of oxygen and metabolites during the anagen phase, when follicular demand peaks. These hypotheses require direct validation in AGA, as markers derived from keloids, systemic sclerosis, or cicatricial alopecia reflect different injury intensities, anatomical sites, and probabilities of stem cell destruction.

## 4 Mechanistic coupling between inflammation and fibrosis

The immune-to-stromal effector arm is discussed first because it provides the immediate link between inflammation and matrix remodeling, although follicular stress is proposed to precede immune activation in this model.

### 4.1 Immune-to-fibroblast signaling

TGF-β is the best-characterized candidate link. Inui et al. found that androgen exposure increases TGF-β1 in dermal papilla cells from balding scalp and that the resulting signal inhibits epithelial growth in co-culture (29). Immune cells could amplify this pathway through region-specific mediators: mast cells can release tryptase, chymase, TNF, IL-6, and TGF-β; macrophage-lineage cells can provide TGF-β, platelet-derived growth factor, IL-1β, TNF, and CCL2; and T cells can contribute cytokine or cytotoxic signals. These mediators may act on perifollicular fibroblasts and dermal sheath cells to alter survival, collagen synthesis, contractility, and protease balance. IL-1β and TNF can induce inflammatory fibroblast programs, whereas IL-6–STAT3 and CCL2–CCR2 signaling can sustain leukocyte recruitment. However, no longitudinal human AGA study has linked a defined immune-cell source and mediator to a specific stromal state and subsequent miniaturization.

Mast cells may rapidly amplify the response via degranulation, whereas monocyte-derived signals are more likely to sustain a slower, repair-like state. Fibroblasts themselves are not a uniform population: cell groups within the papillary layer, reticular layer, perivascular and perifollicular regions, dermal sheath, and dermal papilla differ in both lineage and function. A single mediator might induce collagen production in one cell population while inhibiting hair growth-inducing signals in another. Consequently, measuring total scalp cytokines fails to pinpoint the cellular source or identify the responding stromal compartment—a recurring limitation in the literature.

Overall, regional immune infiltrates and profibrotic mediators are associated features of human AGA, but a causal link between specific immune cells and defined fibroblast states has not been established.

### 4.2 Follicular stress-to-immune signaling

Androgen-sensitive follicular cells provide the best-supported upstream stress signals in the proposed network. DHT-induced DKK1 from balding dermal papilla cells can promote follicular keratinocyte apoptosis (30), and DHT-induced IL-6 suppresses matrix-cell proliferation, inhibits hair-shaft elongation, and promotes experimental regression (31). PGD2 is elevated in balding human scalp and inhibits hair growth through GPR44 (32). These mediators can shorten anagen directly while also altering epithelial barrier function, vascular tone, or immune recruitment. Balding dermal papilla cells show premature senescence associated with p16INK4a (33), and androgen-receptor signaling can accelerate DNA-damage-associated senescence (34). The senescence-associated secretory phenotype, mitochondrial ROS, damage-associated molecular patterns, and oxidized lipids together provide a plausible route from recurrent follicular stress to innate immune sensing.

Recent experiments reinforce a stress–senescence axis, but the conclusions remain model-dependent. A 2026 *Biochemical Pharmacology* study reported that MitoQ upregulates CYP19A1 and protects DHT-exposed dermal papilla cells and an AGA-like mouse model from mitochondrial dysfunction (35). A related 2024 *European Journal of Pharmacology* study from an overlapping author group linked MitoQ/CYP19A1 to WNT/β-catenin signaling and hair growth (36). ACOD1 deficiency has also been associated with DDX1 methylation, mitochondrial dysfunction, and dermal papilla cell senescence in human tissue and experimental systems (37). These studies support follicular stress biology but do not show that mitochondrial dysfunction initiates scalp inflammation in untreated human AGA. Evidence from human AGA tissue and patient-derived models supports DHT-dependent follicular stress and several growth-inhibitory signals, although it remains unclear whether these signals initiate regional immune activation.

### 4.3 Matrix feedback

Once fibroblasts and dermal sheath cells remodel the matrix, the niche could become self-reinforcing. Dense or cross-linked collagen may increase interstitial resistance, distort perifollicular microvessels, and restrict oxygen and nutrient exchange. Hypoxia and lactate can alter macrophage and fibroblast behavior; matrix stiffness can activate integrin–FAK–YAP/TAZ signaling and increase activation of latent TGF-β. Keloid transcriptomics suggest C1Q-associated macrophage–fibroblast signaling (38), systemic sclerosis studies identify macrophage states that balance tissue protection and fibrosis (39), and reviews describe POSTN-positive fibroblast/SPP1-positive macrophage loops (40). These are cross-disease analogies; the corresponding cell states and spatial proximities have not been verified in AGA scalp.

Mechanical feedback also offers a potential mechanism explaining why the disease persists even after the initial androgen signal subsides. A stiffened matrix can expose latent TGF-β, alter integrin clustering, and change cell morphology, while hypoxia enhances oxidative stress and inflammation-associated transcription. Conversely, if collagen cross-linking is limited and the hair follicle stem/progenitor cell compartment remains intact, the removal of DHT might allow for gradual matrix turnover. Whether AGA-associated fibrosis is irreversible remains unknown because treatment-responsive biopsy data are lacking. General skin-fibrosis studies make matrix feedback biologically plausible, but human AGA studies have not established stiffness-dependent persistence or the reversibility of matrix remodeling.

### 4.4 Hair-cycle consequences

The expected outcome is not follicular destruction but a lower probability of robust anagen re-entry. TGF-β, DKK1, PGD2, oxidative stress, and reduced Wnt/β-catenin support can promote premature catagen, reduce matrix-cell proliferation, and impair dermal papilla inductivity. A contracted dermal sheath and stiffened matrix could then constrain dermal papilla volume and communication with stem/progenitor cells. Spatial omics in systemic sclerosis show that transcriptional states depend on tissue location (41), but whether the same spatial dependence applies to AGA scalp remains unknown. The proposed model is bidirectional: follicular miniaturization generates stress signals, while inflammatory stromal remodeling may constrain regenerative capacity. This bidirectional model could account for persistence without assuming that inflammation or fibrosis initiates AGA (**Figure 1**).

**Figure 1:**
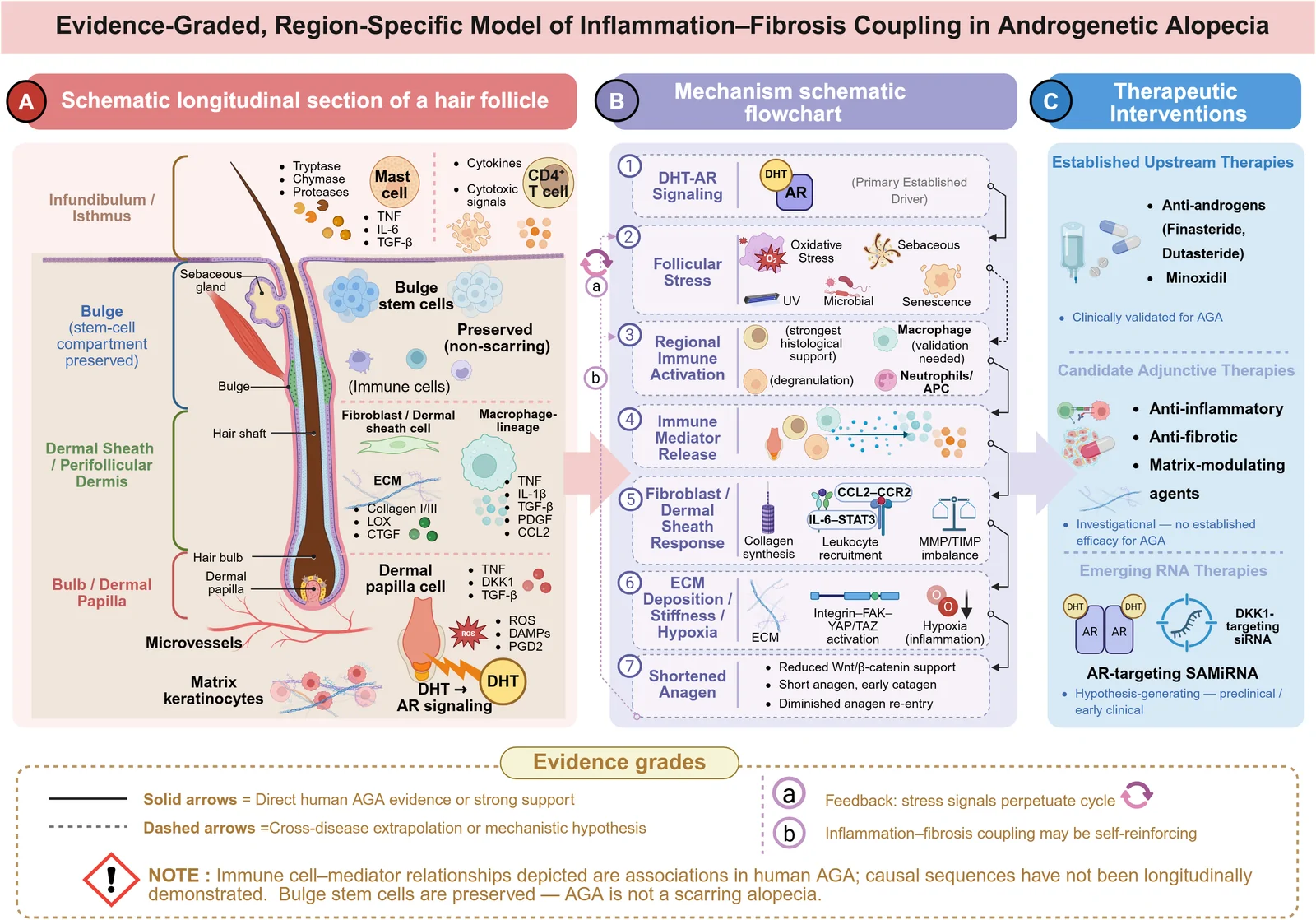
Evidence-graded, region-specific model of inflammation–fibrosis coupling in androgenetic alopecia (AGA). **(A)** maps immune and stromal features to the infundibulum/isthmus, bulge, dermal sheath/perifollicular dermis, and bulb/dermal papilla. **(B)** presents DHT–androgen receptor signaling as the primary established driver. Oxidative, sebaceous, microbial, ultraviolet, and senescence-associated stressors are secondary or proposed contributors, and their proposed link to regional immune activation is shown with a dashed arrow. Candidate mediators from mast cells, macrophage-lineage cells, and T cells connect to perifollicular fibroblast/dermal sheath responses, collagen/CTGF/LOX activity, MMP/TIMP imbalance, stiffness, hypoxia, reduced Wnt/β-catenin support, and shortened anagen. Lowercase labels a and b denote proposed feedback processes within the mechanism schematic rather than separate figure panels: a indicates perpetuation of the cycle by stress signals, and b indicates potentially self-reinforcing inflammation–fibrosis coupling. **(C)** distinguishes established androgen- or growth-directed therapies from candidate adjunctive therapies and emerging RNA approaches. Solid arrows denote direct human AGA evidence or strong support; dashed arrows denote cross-disease extrapolation or mechanistic hypotheses. Arrow style reflects evidentiary support rather than temporal order.

Stages of the hair growth cycle can also confound interpretation. The catagen and telogen phases are normally associated with immune cell migration, vascular regression, extracellular matrix turnover, and dermal papilla repositioning. Consequently, increases in immune or collagen markers observed in cross-sectional analyses may reflect shifts in the distribution of hair cycle stages rather than a stable pathogenic state. Studies should compare hair follicles at the same cycle stage and—after adjusting for the severity of miniaturization—examine whether the same spatial circuits persist.

Shortened anagen and progressive miniaturization are established features of AGA, but current evidence does not isolate a causal contribution of inflammation–fibrosis coupling from that of androgen signaling.

## 5 Biomarkers and spatial evidence

A useful biomarker panel should distinguish active inflammation, matrix remodeling, and established structural restriction. Candidate tissue markers include CD3, CD4, CD8, CD68, mast cell tryptase or CD117, TGF-β pathway proteins, phosphorylated STAT3, COL1A1, COL3A1, fibronectin, α-smooth muscle actin, LOX, periostin, and MMP/TIMP ratios. Trichoscopic findings such as erythema, scaling, peripilar discoloration, white perifollicular structures, and reduced follicular openings can help guide biopsy site selection, though none of these features are mechanistically specific.

Spatially resolved transcriptomic analysis in AGA has linked perifundibular immune infiltration, progenitor cell dynamics, extracellular matrix programs, and epithelial-mesenchymal signatures (42). This approach preserves anatomical context, but the findings require validation in larger independent cohorts using standardized scalp sampling sites. Single-cell and spatial analyses of primary cicatricial alopecias have identified T-cell, macrophage, interferon, and fibrotic pathways targeting the hair follicle (43); meanwhile, paired biopsies in frontal fibrosing alopecia demonstrate that inflammation and fibrosis can extend beyond the clinically apparent borders of involvement (44). These studies identify candidate spatial measurements, but findings from cicatricial alopecia cannot be transferred directly to AGA. Data from multicenter studies on patterned fibrosing alopecia also help define diagnostic boundaries (45).

Biopsy site, hair-cycle stage, sex, disease severity, overlapping seborrheic lesions, and prior treatment can all affect cell counts and matrix measurements. Future studies should pair frontal or vertex samples with occipital scalp, record anagen/catagen ratios, and integrate histology, collagen second-harmonic generation imaging, multiplex immunofluorescence, single-cell RNA sequencing, spatial transcriptomics, and trichoscopy. Because cross-sectional profiling cannot resolve causal direction, treatment-response studies require paired biopsies (Section 1). The occipital scalp is also an imperfect internal control because donor-area miniaturization, diffuse hair loss, aging, or widespread inflammation can reduce regional contrasts. Protocols should document biopsy orientation and depth, follicular density, sebaceous-unit status, distance from clinical margins, and recent topical or systemic therapy. Digital pathology should quantify immune-cell distance from epithelial and stromal compartments rather than use a binary present/absent score. **Table 1** classifies each proposed link by specimen or model source so direct human AGA evidence can be separated from *ex vivo*, patient-derived *in vitro*, animal, and cross-disease support.

**Table 1:** Region-specific immune and fibrotic mediators in AGA, classified by evidence source.

Mediator/cell	Region	Proposed link	Evidence source(s)	Direct human AGA	Experimental/cross-disease support	Marker/target	Major limitation
Mast cells	Infundibulum/isthmus; upper perifollicular dermis	Proteases, TNF, IL-6, and TGF-β may amplify remodeling	Human AGA biopsy + cross-disease support	CD117+ mast cells in selected biopsies	Skin-fibrosis and follicular immune-privilege models	Tryptase/CD117; stabilization	Association does not establish mediator release or causality
T cells	Infundibulum/isthmus; bulb uncertain	Cytokine/cytotoxic effects on epithelium and stroma	Human AGA histology/RNA data + AA models	Upper-follicular CD4+ cells; CD8+/NK signatures	Human/mouse AA; cicatricial-alopecia studies	CD3/CD4/CD8; spatial location	No autoimmune causation; spatial/temporal heterogeneity
Macrophage-lineage cells	Perifollicular and perivascular niches	Candidate cytokine/chemokine and matrix-turnover signaling	Limited human AGA histology + cross-disease support	Macrophage-like cells; no AGA state definition	Keloid/systemic-sclerosis studies	CD68 plus spatial ligand–receptor signatures	High extrapolation risk; AGA states/mediators unvalidated
Dermal papilla/epithelial stress	Bulb/dermal papilla; epithelium	DHT, ROS, DAMPs, and senescence may inhibit growth and recruit immunity	Human AGA tissue + patient-derived *in vitro* + animal	Spatial/stress signatures in human AGA tissue	DHT-exposed patient-derived cells/follicles; AGA-like mice	p16INK4a; mitochondrial/oxidative markers	Model sequence does not prove stress initiates scalp inflammation
TGF-β/CTGF	Dermal papilla, immune cells, fibroblasts	SMAD signaling may increase matrix and suppress epithelial growth	Patient-derived *in vitro* + cross-disease	No direct tissue-level immune-to-fibroblast sequence	Androgen-induced TGF-β1 in balding DPC co-culture; skin-fibrosis models	TGF-β, pSMAD2/3, CTGF; localized modulation	Does not establish the full immune–fibrosis chain
PGD2 and IL-6	Balding scalp and dermal papilla	Growth inhibition, reduced proliferation, premature catagen	Human scalp PGD2 + patient-derived *in vitro*/*ex vivo* + animal	Elevated PGD2 in balding scalp	DPC IL-6; follicle/mouse experiments	PGD2/GPR44, pSTAT3; pathway blockade	Growth inhibition supported; fibrosis and causal primacy untested
Fibroblasts/dermal sheath	Perifollicular matrix; connective tissue sheath	Contractility and collagen/fibronectin may restrict crosstalk	Human AGA histology/single-cell + model support	Fibrosis histology; contracted sheath state	AGA organ and cross-disease fibrosis models	COL1A1/3A1, α-SMA, periostin	States, reversibility, and androgen-independent effects remain unclear
LOX, MMP/TIMP, stiffness	Remodeled extracellular matrix	Cross-linking and altered turnover may reinforce mechanosignaling/hypoxia	Cross-disease/model only	No longitudinal human AGA validation	Keloid/skin-fibrosis mechanisms	LOX, MMP/TIMP ratio, collagen imaging	High extrapolation risk; AGA relevance/reversibility unknown

Direct human AGA evidence denotes clinical scalp specimens; other support is labeled as patient-derived *in vitro* or *ex vivo*, animal, or cross-disease evidence.

## 6 Therapeutic implications

Established androgen- and growth-directed therapies remain the foundation of AGA treatment. Minoxidil and finasteride remain standard options, and dutasteride is an important androgen-targeted option whose approval and applicability vary by sex and jurisdiction (46, 47). By lowering DHT, anti-androgen therapy may also attenuate androgen-dependent TGF-β, DKK1, IL-6, and cellular stress signaling, whereas minoxidil supports hair growth and vascular function. Trials rarely include paired immune or matrix endpoints, however, so improved hair counts cannot be assumed to indicate reduced fibrosis. The clinical value of this framework would lie in identifying persistent inflammatory or stromal changes that limit recovery despite adequate androgen control.

Three provisional endotypes could guide future studies of patient selection and treatment timing. An inflammation-predominant endotype could combine perifollicular erythema or scale with spatially localized CD3/CD4 cells, mast-cell tryptase/CD117, or pSTAT3; such patients might be candidates for a biomarker-selected anti-inflammatory adjunct after standard therapy. An active-remodeling endotype could show procollagen, fibronectin, periostin, α-smooth muscle actin, LOX activity, or an altered MMP/TIMP ratio and might justify trials of localized, reversible matrix modulation. An established structural-restriction endotype could show dense cross-linked collagen and persistent sheath contraction, predicting lower reversibility and favoring earlier intervention rather than more aggressive systemic therapy. These categories remain research constructs, not validated clinical diagnoses.

Candidate adjuncts include topical anti-inflammatory treatment, mast-cell stabilization, PGD2–GPR44 blockade, senescence modulation, and localized regulation of TGF-β/CTGF, LOX, JAK–STAT, or integrin–FAK–YAP/TAZ signaling. They remain hypotheses rather than clinical recommendations, and broad immune or TGF-β inhibition could impair surveillance, wound repair, and normal cycling. RNA-based approaches could target upstream components of the proposed pathway. Topical SAMiRNA targeting androgen-receptor mRNA reduced AR expression in human follicular models and was evaluated in randomized double-blind clinical studies (48), whereas cholesterol-modified DKK1 siRNA promoted hair-regrowth signals in mice (49). AR silencing targets the primary androgen pathway, while DKK1 silencing may restore Wnt support; neither has yet shown that an inflammatory–fibrotic endotype is reversed. Delivery depth, durability, off-target activity, innate immune stimulation, and long-term safety remain important unresolved concerns. The distinction among established, candidate adjunctive, and emerging RNA-based approaches is summarized in **Figure 1C**.

The same framework may also inform the evaluation of procedural and regenerative therapies. Microneedling combined with standard therapy can improve density or diameter, but protocols and adverse events are heterogeneous (50). PRP evidence is limited by small samples, variable preparations, and risk of bias (51), while laser or LED trials also show heterogeneity (52). These interventions may affect repair, vascular, or immune pathways, but increased hair count does not establish fibrosis reversal; broader regenerative reviews likewise emphasize insufficient standardization (53). Timing may matter: anti-inflammatory adjuncts are most plausible during active immune and matrix turnover, whereas matrix-directed strategies should be tested only after androgen-driven stress is controlled. Excessively deep or frequent wounding could aggravate inflammation.

A biomarker-enriched, sequential trial could test this framework. Patients would first receive optimized anti-androgen or growth-supportive therapy; those with persistent, spatially confirmed inflammation or active remodeling would then be randomized to a targeted adjunct. Outcomes should pair hair count and shaft diameter with collagen architecture, immune-cell localization, pathway pharmacodynamics, adverse events, and durability after treatment cessation. Prespecified scalp regions, hair-cycle context, administration depth, and stopping criteria are essential. Given the chronic, non-life-threatening course of AGA, topical delivery and reversible pathway modulation are more appropriate initial strategies than systemic antifibrotic therapy. This design could test whether a defined endotype modifies treatment response rather than merely correlating with disease severity.

## 7 Conclusions and future directions

AGA remains genetically influenced and androgen dependent, but miniaturization occurs within an immune, vascular, and stromal microenvironment. Human histology and emerging omics identify perifollicular inflammation, mast-cell and lymphocyte signals, connective tissue sheath changes, and fibrosis in subsets of patients (7, 9, 10). Patient-derived cells and follicular models support links involving TGF-β, IL-6, DKK1, PGD2, oxidative stress, mitochondrial dysfunction, and senescence. Findings from other fibrotic disorders suggest how these signals could alter fibroblast states and tissue mechanics, but the proposed immune-to-stromal circuits require spatial and longitudinal confirmation in human AGA.

It remains unclear whether inflammation precedes miniaturization, follows follicular stress, or participates in a bidirectional process, and whether matrix remodeling limits recovery or merely records chronic turnover. Resolving this question requires longitudinal, trichoscopy-guided paired biopsies linked to treatment response, with spatial transcriptomics, multiplex imaging, quantitative collagen analysis, and immunocompetent follicular models. Trials should test whether standard anti-androgen therapy changes immune or matrix features, whether early remodeling is reversible, and whether validated inflammation-predominant or remodeling-predominant endotypes benefit from localized adjuncts without disrupting normal repair.

Current evidence supports DHT-dependent signaling as the primary mechanism of AGA. Regional microinflammation and fibrosis are reproducible in some patients, but inflammation–fibrosis coupling is better regarded as a testable modifier of disease persistence and treatment response than as an established universal cause.
